# Pangenomics in Microbial and Crop Research: Progress, Applications, and Perspectives

**DOI:** 10.3390/genes13040598

**Published:** 2022-03-27

**Authors:** Sumit Kumar Aggarwal, Alla Singh, Mukesh Choudhary, Aundy Kumar, Sujay Rakshit, Pardeep Kumar, Abhishek Bohra, Rajeev K. Varshney

**Affiliations:** 1 ICAR-Indian Institute of Maize Research, PAU Campus, Ludhiana 141004, India; sumit.aggarwal009@gmail.com (S.K.A.); allasingh.panesar@gmail.com (A.S.); mukesh.choudhary1@icar.gov.in (M.C.); pardeepkumar656@gmail.com (P.K.); 2 School of Agriculture and Environment, The University of Western Australia, Perth, WA 6009, Australia; 3 The UWA Institute of Agriculture, The University of Western Australia, Perth, WA 6009, Australia; 4 ICAR-Indian Agricultural Research Institute, New Delhi 110012, India; kumar@iari.res.in; 5 ICAR-Indian Institute of Pulses Research (IIPR), Kanpur 208024, India; abhi.omics@gmail.com; 6 State Agricultural Biotechnology Centre, Centre for Crop and Food Innovation, Food Futures Institute, Murdoch University, Murdoch, WA 6150, Australia

**Keywords:** pangenome, biological research, genome sequence, germplasm, novel genes, evolution, NGS

## Abstract

Advances in sequencing technologies and bioinformatics tools have fueled a renewed interest in whole genome sequencing efforts in many organisms. The growing availability of multiple genome sequences has advanced our understanding of the within-species diversity, in the form of a pangenome. Pangenomics has opened new avenues for future research such as allowing dissection of complex molecular mechanisms and increased confidence in genome mapping. To comprehensively capture the genetic diversity for improving plant performance, the pangenome concept is further extended from species to genus level by the inclusion of wild species, constituting a super-pangenome. Characterization of pangenome has implications for both basic and applied research. The concept of pangenome has transformed the way biological questions are addressed. From understanding evolution and adaptation to elucidating host–pathogen interactions, finding novel genes or breeding targets to aid crop improvement to design effective vaccines for human prophylaxis, the increasing availability of the pangenome has revolutionized several aspects of biological research. The future availability of high-resolution pangenomes based on reference-level near-complete genome assemblies would greatly improve our ability to address complex biological problems.

## 1. Introduction

The initiation of the human genome sequencing project in 1990 served as a breakthrough in biological sciences. It opened the way for many new scientific domains including the study of proteome and metabolome, etc. The genome sequencing efforts have since been extended to many other organisms, including bacteria, fungi, plants, and animals. The breakthroughs in DNA sequencing have led to a considerable reduction in time and cost required for decoding the complete genome sequence [1,2,3]. This has been reflected in a deluge of genome sequencing datasets that have been deposited in public databases [4]. The increasing availability of a large number of genomes boosted the comparative genomics studies for the estimation of genetic variation among the individuals of a species. Growing realization about the inadequacy of a single reference genome to catalogue entire gene content of a species stimulated interest on sequencing multiple genomes, resulting in the development of the pangenome concept [5]. Pangenome analysis has the potential to serve as a game-changing approach for covering entire species diversity using advanced sequencing platforms [5,6]. Analysis of genomes of six strains of *Streptococcus agalactiae* laid the foundation of developing a pangenome that has two major components: core genome represented by the fixed genome portion present in all six strains (constituting up to four-fifth proportion in any single individual genome) and the remaining one-fifth “variable” portion corresponded to strain-specific genes, often designated as “dispensable or accessory” [7]. The variable genomic portion can be classified into two distinct parts-unique genes (restricted to one individual only) and dispensable genes (genes common across at least n−1 individuals or absent in some individuals). The core and variable genes signify the essentiality and the diversity of the species, respectively [8]. The development of pangenomes has provided researchers with new tools and approaches to find novel genes and understand how the genome shapes the diversity of an organism. Newly constituted pangenomes throw light on many aspects of basic and applied sciences, including evolution, design of vaccines, and antibacterial.

In the case of crops, wild relatives and diverse germplasm have contributed immensely to domestication and systemic improvement, resulting in the development of modern cultivars. Accounting for the broad genetic diversity contained in wild species in a particular genus extends the pangenome concept from species level to genus level [9]. Super-pangenome, a pangenome of pangenomes, encompasses different wild species in the given genus and hence expands the possibility to harness the maximum genome diversity available in the particular genus. Another remarkable improvement led by the availability of pangenome is the cataloging of large structural variations (SVs) in the genomes [10]. The present article highlights the importance of SVs and discusses the emergence and subsequent maturation of the pangenome concept and its growing applications in diverse fields of biology and plant science.

## 2. Pangenome: Concept and Types

The term “pangenome” (“pan” in Greek-means “whole”) describes the total of essential genes in a complete genome dataset of the given species [5].

**Figure 1:**
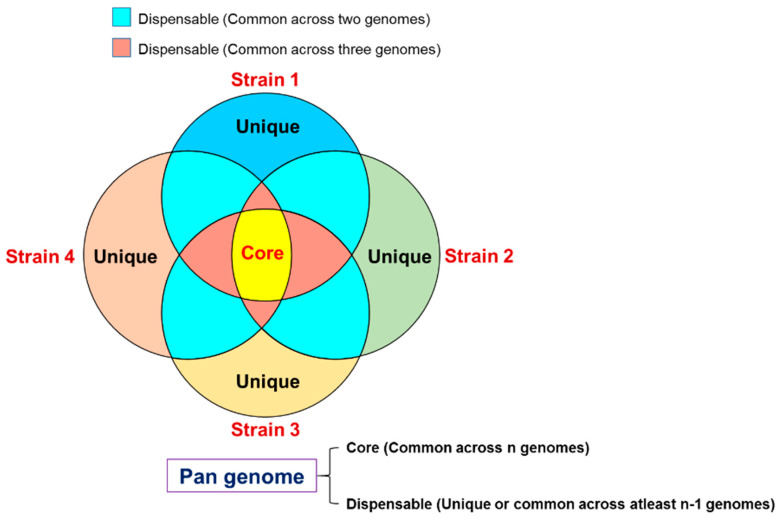
Organization of a pangenome composed of core and dispensable components of the genome.

The pangenome comprises of three parts: (i) Core genome, formed by genes shared by all genomes and usually involved in essential cellular processes; (ii) accessory or dispensable genome, composed of genes absent in some isolates; and (iii) species-specific or strain-specific genes, genes restricted to the single genome [5,7]. Dispensable and species/strain-specific genes correspond to the variable part of the genome. Thus, pangenome can be constructed by the identification of core and variable genes using the genome of particular individuals or strains of any species (Figure 1). Genes comprising the accessory and species or strain-specific genome are often, but not always involved in the adaptation of an organism to a particular niche. The core genome is highly conserved and involved in basic biological processes like replication, translation, and cellular homeostasis. Dispensable/accessory genome subset of genes emerge by horizontal gene transfer shared between some organisms (but not present in all organisms under study and much more common in prokaryotes compared to Eukaryotes) and hence are associated with specific functions like survival, virulence, or resistance to antibiotics [11]. The accessory genes are under mutational pressure which likely gives rise to new alleles for better adaptation to a particular niche. In contrast, the core genome is under strong selective (or evolutionary) pressure and hence highly conserved. Species-specific/strain-specific genomes are present in a single species that emerged by horizontal gene transfer at the inter-species level, whereas strain-specific genes are only present in one strain and are at the intra-species level and associated with the pathogenicity of a particular strain. Conclusively, core and accessory parts of the genome drive the pangenome diversity.

**Figure 2:**
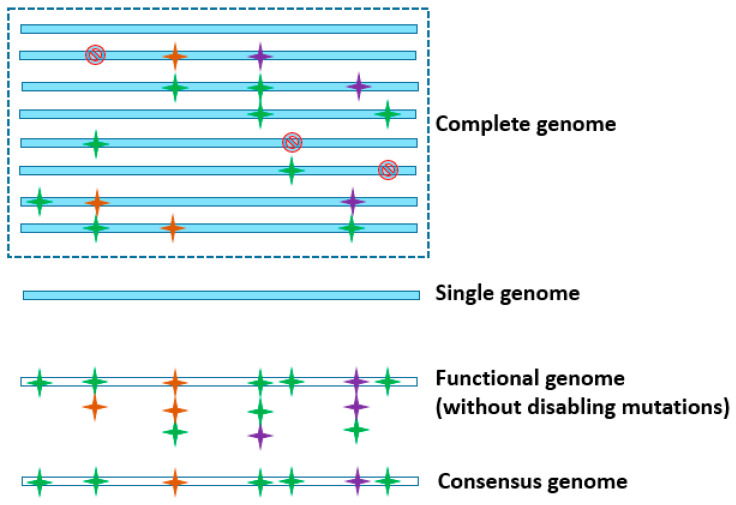
Different forms of a reference genome. The horizontal bars represent the DNA sequence of a genome. 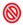 represents a disabling mutation that disrupts the gene function. 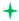, 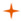, and 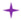 depict various sequence polymorphisms.

An accurate reference genome sequence is an important resource for understanding the biological functions using NGS-based approaches. However, considering the inability of a single individual to represent entire genetic diversity, researchers soon realized the need to look beyond a single reference genome via utilizing the available or generating additional sequence information on multiple genomes. The Computational Pangenomics Consortium [12] notes four types of genomes, *viz.*, complete genome, which consists of all the sequences ever known for an organism; genome of a single individual; functional genome, lacking the disabling mutations known for a genome; and, consensus genome, based on the consensus of available sequence data (Figure 2). The choice of a “reference genome” depends on the objectives and the resource availability. In the post-NGS era, an increase in large-scale genome sequencing projects and the quest to explain hitherto unknown mechanisms have placed more emphasis on a “pangenome” as a new reference, to better understand the genetics of organisms.

Pangenome is classified as “*open*” or “*closed*”, depending on the number of new genes added per genome sequenced [5]. If with the addition of a new genome sequence, the number of newly discovered genes keeps on increasing, the pangenome is said to be “open” and warrants further sequencing. On the other hand, if the number of new genes discovered remains the same upon sequencing of new genomes, the pangenome is referred to as “closed”. Exemplified by *S. agalactiae* (group B streptococcus; GBS), the addition of sequencing information of new strain led to expanding the pangenome volume by 33 novel genes. For example, an open pangenome was noticed in the case of five strains of *S. pyogenes* exhibiting similar genomic diversity but contributing to the expansion of pangenome by 27 specific genes for the addition of each novel genome [5]. In another study, eight independent *Bacillus anthracis* isolates were sequenced but the pangenome volume expansion via a rise in the number of novel genes halted after the addition of sequence information of only four genomes [5]. Therefore, the *B. anthracis* species is considered to be an example of a “closed” pangenome as only genomic information of four isolates is good enough to represent the entire genomic content of this species. Recent research has reported the development of closed pangenomes in various crop species including rice [4,13,14,15,16].

## 3. Importance of Pangenome

The genomic era started over a decade ago, but still, bacterial species have not been explored to a larger extent. The sequencing studies of multiple strains in some species revealed the possibility of finding novel genes with the inclusion of sequencing information of each additional strain. Later, mathematical modeling [17] also supported this fact of discovering novel genes in some species even after the inclusion of hundreds of genomes per species. Therefore, a need was felt to discover a more accurate way to explaining the entire genetic information of bacterial species. Considering that the pangenome of any organism contains the highest amount of genetic information compared to a single genome, changes at the pangenome level may help understand the symptoms and infection in the host [8].

## 4. Structural Variations Are Crucial for within-Species Diversity

**Figure 3:**
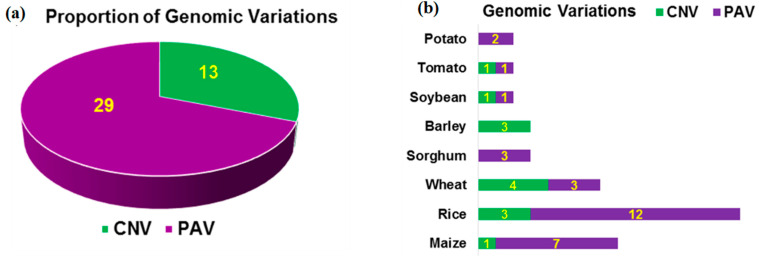
Genomic variation in terms of proportion (**a**) and distribution (**b**) of PAVs and CNVs in the genome of major crops for agriculturally important traits (Interpreted from Tao et al. [21].

The improvement of any organism depends upon the existing genetic variation. The genetic variation for the agronomic traits among individuals of the same species or different species is caused by the differences in the sequence of nucleotides or bases called sequence variations and large-scale (usually >1 kb) DNA rearrangements referred to as structural variations (SVs). These SVs arise from various mechanisms like recombination, double-strand break repairs, and transposable elements and range from few base pairs to several megabases. The large SVs can be of two types: (i) copy number variation (CNV), defined as the variable number of copies of a particular sequence among different individuals and (ii) the presence-absence variation (PAV) created by the absence of a particular sequence in few individuals which otherwise exists in rest of the individuals [10,18]. Hence, PAV can be considered as an extreme form of CNV where one particular sequence is completely absent in a few individuals. Unlike humans, the abundance of CNVs has been reported from the majority of crop species and hence is considered to assume greater significance for causing variation in trait expressions [19]. One of the key objectives of the pangenome analysis is to capture genome variations caused by the large SVs including PAVs and CNVs. Generally, plant disease-associated defense genes are known to display CNVs [20]. Recent research supports a greater role for PAVs than CNVs in shaping crucial plant phenotypes [21] (Figure 3a). The role of PAVs in stress response and domestication traits including shattering, photoperiod sensitivity, and male sterility has been evident in major crops such as rice, maize, and sorghum [22,23,24]. In plant genomes like maize that are characterized by extensive repetitive DNA sequences, the presence of transposable elements (TEs) could explain the abundance of PAVs [19,25]. By contrast, most of the agronomically important traits are governed by CNVs in barley (Figure 3b). Hence, variable distribution of SVs among crops could be the reason for the prominence of PAVs or CNVs in a particular crop. In rice, the SVs are reported to influence gene expression and their distribution among the populations helps understand the domestication process [26].

## 5. Pangenome Construction: Basic Approaches and Critical Factors

**Figure 4:**
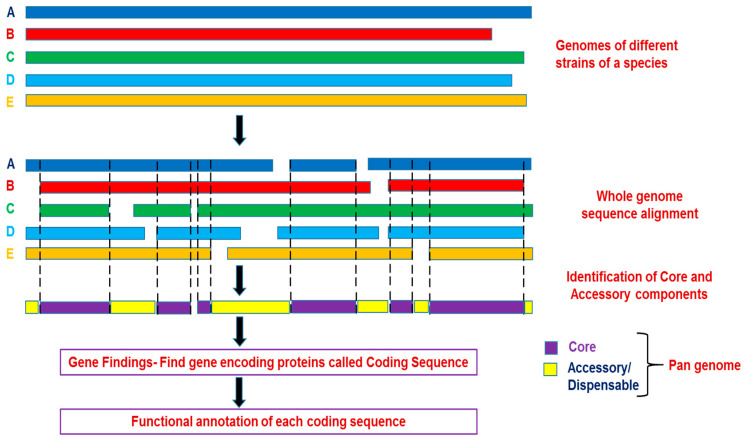
A basic approach for pangenome construction. Genome sequences of different strains represented schematically as A (**blue**), B (**red**), C (**green**), D (**light blue**), and E (**yellow**) are aligned to identify the core and accessory components of the pangenome.

Pangenomes can be generated by various approaches such as the comparative de novo approach [13,27], an iterative assembly approach [14,16,28,29], and the “map-to-pan” approach [30]. Further, [31] summarized the current approaches for construction pangenome in plants. Figure 4 illustrates the general steps for pangenome construction. It includes the genome assembly of the different strains followed by genome alignment and identification of core and dispensable parts of genomes. The identified genes are then used for functional annotation. The different approaches of pangenome construction rely on this basic procedure with slight modifications. The comparative de novo approach, as exemplified by initial pangenome studies in crops like rice [27], soybean [16], relies on the principle of comparison of annotations of de novo genome assemblies of individuals for identification of core and dispensable genes, whereas the rest two approaches rely on building a pangenome reference sequence. Then, the identified pangenome sequences are annotated. Finally, the genic PAVs are identified via aligning the mapping reads on the pangenome. However, iterative assembly and map-to-pan approaches follow different strategies for the construction of a pangenome sequence as the former uses mapping reads from initial samples to align with whole-genome assembly reference accompanied by reference assembly update by addition of unmapped reads [6]. In contrast, the later approach starts with de novo assembling of individual genomes followed by the use of the reference genome to map low-quality de novo assemblies to construct pangenome [30]. The two approaches have been used in the recent pangenome studies in crop plants based on large-scale genome sequencing of more than 3000 accessions (iterative assembly approach: [4]; map-to-pan approach [32]). The benefits of iterative assembly and “map-to-pan” approaches are low sequencing depth-based identification of genic PAVs via mapping short reads to an annotated genome but with limited applicability to simple genomes with less repetitive gene sequences [33,34].

The recent development of long-read sequencing or the third-generation sequencing platforms such as Pacific Biosciences (accessed on 16 March 2022)(PacBio) and Oxford Nanopore Technologies (accessed on 16 March 2022)(Nanopore) is likely to relieve the current drawback of the high-cost associated with the comparative de novo approach, thus greatly enhancing the utility of this approach in pangenome analysis. Alternatively, skim-sequencing can be used for pangenome construction via sequencing multiple varieties and assembling reads that do not align to the reference genome, especially for simpler genomes as it fails to effectively capture SVs in complex genomes [35].

The factors that critically influence the pangenome analysis include the quality of the reference assembly, its annotation quality, orthologous gene detection, selection of appropriate individuals, and suitable pangenome analysis tools or software [6]. The reference genome assembly should be of sufficient quality in terms of its size, completeness, and fragmentation level to facilitate better quality annotation. The long-read sequencing technologies could overcome the problem of fragmented assemblies resulting from the inability of short-read sequencing technologies to resolve the repetitive sequences in complex genomes [36]. The fragmented assemblies cause under-prediction of the total number of genes and also affect the detection of SVs, hence resulting in the poor quality functional annotation. The completeness of genome assemblies can be assessed by using several metrics like Core Eukaryotic Genes Mapping Approach accessed on 16 March 2022) CEGMA) [37] and Benchmarking Universal Single-Copy Orthologs accessed on 16 March 2022 (BUSCO) [38]. Another important factor concerns the selection of candidate individuals for pangenome construction. The candidate individuals should be highly diverse and optimum in number because less diverse and low population size of candidate individuals downgrades the representation quality of pangenome [6]. The optimum number of candidate individuals for a pangenome study can be decided by using the modelling of pangenome expansion and core genome reduction [7].

Mapping and assembling of genes are important issues to consider in the pangenome analysis. Various methods have been reviewed [39]. A pan reference for anchoring additional genes can be created via different approaches. One approach is using the synteny-based co-localization of core genes adjacent to the dispensable genes. Another is anchoring dispensable genes by using genetic marker-based linkage between core and dispensable genes. Alternatively, sequence-similarity-based approaches can be used for anchoring. However, repeat sequences pose a challenge in this case.

## 6. Software’s/Tools for Pangenome Analysis

Software packages and tools are very important to categorize orthologous genes, calculate pangenomic profiles, integrate gene annotations, and construct phylogenies [40]. A detailed description of the various features of different software used in the pangenome analysis has been provided in Table 1. PanSeq (Pangenome Sequence Analysis Program) accessed on 17 September 2021 (Public Health Agency of Canada, Lethbridge, AB, Canada) is an online platform for the identification of core and accessory genomic regions. As a web server, it is platform-independent and makes use of NCBI resources. Similarly, PanFunPro, accessed on 17 September 2021 (Technical University of Denmark, Kongens Lyngby, Denmark) (Pangenome Analysis Based on Functional Profiles) is a tool for pangenome analysis using functional domains from HMM (Hidden Markov Models). GET_HOMOLOGUES is used to perform comparative-genomic analysis of bacterial strains and to build clusters of orthologous groups. ITEP accessed on 17 September 2021 (University of Illinois at Urbana-Champaign, Urbana, IL, USA) (Integrated Toolkit for the Exploration of Microbial Pangenomes) software system has been developed to predict protein families, orthologous genes, functional domains, pangenome (core and variable genes), and metabolic networks for related microbial species. PanGP accessed on 17 September 2021 (Beijing Institute of Genomics, Chinese Academy of Sciences, Beijing, China) (Pangenome Profiles) is a tool for a quick analysis of the bacterial pangenome using a large number of strains. PGAP accessed on 17 September 2021 (Beijing Institute of Genomics, Chinese Academy of Sciences, Beijing, China) (Pangenome Analysis Pipeline) is developed to perform pangenome analysis, genetic variation, evolution, and function analysis of gene clusters. PGAT accessed on 17 September 2021 (University of Washington, Seattle, WA, USA) (Prokaryotic Genome Analysis Tool) is a web tool to compare multiple strains of the same species, to predict genetic differences. Its analyses include pangenome, synteny, identification of genes present or absent in a dataset, comparison of sequence variants in orthologous genes, comparison of genes in metabolic pathways, and improvement of functional annotation. EDGAR accessed 17 September 2021 (Bielefeld University, Bielefeld, Germany) (Efficient Database Framework for Comparative Genome Analyses Using BLAST Score Ratios) is a webtool that performs orthology analysis to calculate pangenome, core-genome, and singletons are computed using BLAST Score Ratio Values (SRV). This method divides the BLAST bit score by the maximum possible bit score, generating the SRV and the cutoff is calculated using a sliding window instead of a fixed SRV threshold of 30, as proposed by [41]. Micropan accessed on 17 September 2021 (Norwegian University of Life Sciences, Norway) package is another tool that helps in pangenome and associated analysis. SplitMem accessed on 17 September 2021 (Stony Brook University, Stony Brook, NY, USA) is a graphical software for producing a compressed colored graph of the pangenome.

## 7. Applications of Pangenomics in Biological Research

The concept of pangenome started with bacterial species and was extended to other organisms, including crops later on with diverse applications. Pangenomics has facilitated applied research directly in some cases by identifying industrially relevant microbial resources and fostering the design of vaccines. It has helped in the identification of novel genes for agriculturally important traits in different crops. In the following section, the role of pangenomics in advancing the basic biological research eventually leading to real-life applications has been discussed.

### 7.1. Finding Novel Genes

The advent of massively parallel sequencing at relatively cheaper costs has facilitated the large-scale generation of genome sequence information. However, computational algorithms are required to derive meaningful inferences from these huge datasets. The urgency of robust algorithms is greater for pangenome studies, as it does not discard any data, rather attempts to map the DNA sequences obtained to relevant genomic locations in already sequenced strains. Pangenomic comparison often relies on the relationship of homology between newly generated DNA sequences and those already available in the repositories. The genes arising from a speciation event, are termed orthologs whereas paralogs result originate from DNA duplication events. Bosi et al. [80] have reviewed the concept of homology, particularly orthology, for data mining of pangenomic sequences. The authors describe Bidirectional Best Hits (BBH) as a simple and fast approach to identifying orthologous genes. This approach relies on the assumption that orthologous genes are more similar to each other than they are to any other sequences in the genome. Databases like Clusters of Orthologous Groups of proteins (COGs) (accessed on 16 March 2022) and Kyoto Encyclopedia of Genes and Genomes (KEGG) (accessed on 16 March 2022) are used to define orthology relationships and categorize pangenome into the core- and accessory-genomes. Pangenomics adds information to public sequence repositories. Novel genes, elucidated by pangenomic analysis have high potential in biotechnology applications. Othoum et al. [81] describe the use of pan-genomic analysis to mine genetic regions capable of biosynthetic capabilities in *Virgibacillus* strains. The novel biosynthetic capabilities carry industrial importance, especially as pharmaceuticals. The study revealed the involvement of genes encoding for protein classes like non-ribosomal peptide synthetases (NRPS), polyketide synthetases (PKS), ribosomally synthesized and post-translationally modified peptides (RiPPs), etc., in anti-tumor, antimicrobial and immunosuppressive properties. Analysis of nine *Virgibacillus* strains showed that most genes encoding for NRPS are present in genomic islands, predicted to have been transferred by lateral DNA flow. The authors deciphered two strains, *V. dokdonensis Bac330* and *Virgibacillus Bac332* to be important and containing more modular genes as compared to other species. The two strains being isolated from Red Sea mangrove mud may have attained a higher proportion of biosynthetic capabilities as a result of the potential environmental stress encountered. Hence, apart from elucidating novel genomic islands, gene findings also led to the identification of potential industrially important strains. In another study, ref. [82] demonstrated the potential of two more Red Sea strains *B. paralicheniformis* (*Bac48*) and *B. halosaccharovorans* (*Bac94*), which are capable of secreting twice as much protein as the model strain *B. subtilis* 168. The strain *Bac94* was shown to be enriched with genes associated with the Tat and Sec protein secretion system, hence making it a promising system for recombinant protein expression work.

### 7.2. Revealing Niche-Specific Fitness

The genes in the accessory genome are often linked to traits that influence an organism’s ability to migrate to a new niche. For example, unlike autochthonous organisms that colonize the intestine permanently, some lactobacilli are not capable of permanently residing in the intestine. These bacteria then reside in the gut for a shorter time as commensals. The organisms in a commensal relationship neither harm nor benefit each other. *Lactobacillus rhamnosus* is a good example of commensalism. It is used as a pro-biotic and has great potential in functional foods. Implications of surface-exposed proteins for niche-specific fitness were evident in *L. rhamnosus* based on a pangenomic analysis using genomes of 13 strains isolated from various origins [83]. An operon spaCBA that encodes SpaCBA-called pili has been implicated to be essential for niche adaptability of *L*. *rhamnosus* strains. The pilli enables the microbe to have a mucoadhesive phenotype. This phenotype is a rare and uncommon occurrence in *L. rhamnosus*. The above finding also explains why some strains can adapt to particular niches better than others.

Pangenomics analysis can elucidate genes that impart niche specificity. McInerney et al. [84] have argued that extensive pangenomes in prokaryotes are the result of adaptive evolution, which contributes to the fitness of an organism. By linking organism’s lifestyle with the proportion of the core genome in the pangenome, the authors presented a perspective that most of the accessory genes appear to confer capabilities that are advantageous for the fitness of the organism. However, ref. [85] contested the above notion. The authors argued that accessory genes could have deleterious effects. As such, the accessory genome is not composed of genes that only confer a fitness advantage. For accessory genes with deleterious effects, there is a selection to lower the uptake. Such genes, even when taken up, are consequently lost from the genome. Earlier, ref. [86] argued that gene loss events could lead to an underestimation of the core genome in some cases. Together, the above studies indicate that niche fitness is not the only function of the accessory genome. Livingstone et al. [87] studied the pangenome of *Corallococcus*, which is an abundant genus of predatory soil myxobacteria. Its accessory genome was found to encode for proteins that are involved in predatory defense mechanisms or the generation of secondary metabolites. This also makes the genus a promising candidate for novel bioactive compounds with antimicrobial properties like corallopyronin, corallorazine, and coralmycin. Pangenome serves a broad role, including host-pathogen interactions and predation in microbes, speciation, and contributing to domestication and heterosis in the case of plants.

### 7.3. Evolution, Domestication and Breeding History

Crop domestication commenced around 10,000 years ago in the Fertile Crescent. Attempts to modify wild crops according to human needs have led to marked changes in the crucial plant phenotypes, referred to as domestication traits. Evidence suggests that crop domestication has been associated with trade-offs that reduce the fitness of the crops due to the accumulation of deleterious genetic variations. Availability of the sequence information on multiple genomes provides an enormous opportunity to refine the crop domestication and breeding history. For instance, a large-scale analysis of the genome-wide diversity patterns and domestication-associated loci in rice suggested the first domestication of *Oryza sativa* ssp. *japonica* from the *O. rufipogon* whereas the *O. sativa* ssp. *indica* resulted from the cross involving *japonica* and local wild rice [88]. Zhang and colleagues [89] studied 10 species of poplar to understand their evolutionary history. The authors found substantial DNA variations between the species and reported that the major differences among the poplar species were attributed to R genes for disease resistance with loss-of-function mutations, and the genes for self-incompatibility. Due to the comprehensive coverage of the genome of a particular species, pangenomics is a promising tool for phylogenetic analysis to understand evolutionary dynamics, as exemplified by a recent pangenome analysis to understand eggplant domestication [90]. For studies on evolution, a key question has been to evaluate the number of genomes to sequence for consideration in the analysis of evolution. Bacteria tend to have open pangenomes due to higher gene flow between them. Several pangenomes in different crops have been constructed, and were enlisted in recent studies. [77,91,92,93,94] reviewed the relation between pangenome, machine learning, and genomic selection in plants.

### 7.4. Elucidating Host-Pathogen Interactions

Pangenomics has been used to understand the genes coding for the pathogenicity repertoire of pathogens and how they interact with host systems. Hu et al. [95] describe that comparative genomics has been used to understand strain-to-strain variation and estimate differences between pathogens and their near neighbor, non-pathogenic organisms. The interaction between host and pathogen is ever-evolving, with both adapting ways to ensure their survival in the antagonistic interaction. An open pangenome, where a species can acquire new genes, which could, among other factors, be due to its particular lifestyle, carries the potential to influence host–pathogen interactions in novel ways. DNA transfers and inherent genomic diversity can both lead to an increase in the repertoire of genes responsible for pathogenesis. Casa-Esperón et al. [96] have reviewed the role of horizontal DNA transfer in the evolution of host–pathogen interactions. Perna et al. [97] compared non-pathogenic *Escherichia coli* strain K12 with pathogenic *E. coli* O157: H7. The authors concluded that the phage-mediated horizontal flow of DNA was responsible for the pathogenicity of *E. coli* O157: H7. Pangenomic analysis [98] has concluded that considerable genomic diversity exists between *E. coli* species, besides phage-mediated transfers. Analysis of 17 *E. coli* strains revealed that in *E. coli*, while the core genome consists of approximately 2200 genes, the accessory or dispensable genome consists of about 13,000 genes. Thus, at the time of the study, *E. coli* dispensable genome represented a staggering proportion (~83%) of its pangenome. Hence, pangenomics analysis is essential to understand the different ways by which an organism, especially with an open pangenome, can interact with its host. Badet et al. [99] studied the pangenome of the fungal pathogen of wheat, *Zymoseptoria tritici*, taking 19 samples from six continents. Major chromosomal rearrangements that include presence/absence variation were observed in the fungal strains. The authors reported that the dispensable genome contains pathogenesis-related genes, which encode proteins responsible for plant tissue degradation and manipulation of host functions. Plissonneau et al. [100] also reported that in *Z. tritici*, the dispensable genome largely accounts for its adaptive evolution. A similar study identified pangenome for host–pathogen in *Pantoeastewartia* subsp. *indologenes* (Psi) and foxtail millet and pearl millet [101]. This way, pangenomics add a new dimension to the study of host–pathogen interactions by moving beyond the historical events of lateral DNA transfer and along with the former, focusing also on the pan-genetic complement for understanding essential genes related to pathogenicity.

### 7.5. Explaining Heterosis

Large SVs influence many phenomena including metabolism, flowering, nutrient use efficiency, and stress response [27,102,103,104]. It was earlier hypothesized that CNVs and PAVs may not result in large phenotypic differences as many genes in plants are organized in the form of a gene family [105]. Hence, there is “partial redundancy of the function”, whereby loss-of-function or altered function resulting from CNVs/PAVs in one gene would be partially offset and compensated by other genes of the family.

**Figure 5:**
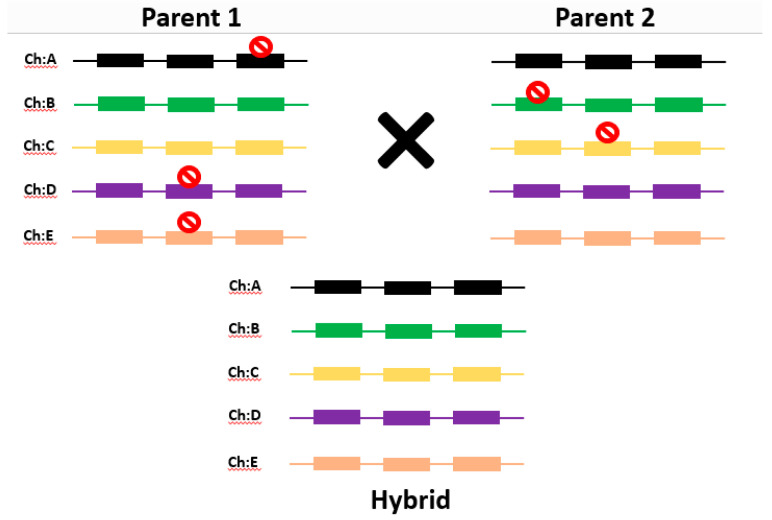
A model of heterosis proposed by Swanson-Wagner et al. [105]. Bars represent genes. Three genes are considered in each hypothetical gene family, situated on different chromosomes. 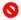 represents “functional block” leading to null or altered protein function. In a real scenario, accumulation of a similar effect with many gene families leads to reduced vigor in inbreeds and heterosis in hybrid. Pangenomics can help to unravel heterosis in a phenotypic trait by discovering new gene variants.

Given this understanding, gene function was conceptualized in the form of a “functional block”, whereby each gene product contributes a certain function to the concerned phenotype. The authors explained that although gene function loss in one family may not result in much difference, loss or alteration of function of some genes in many gene families can lead to decreased vigor. In a hybrid, this effect will be partially nullified, explaining the “hybrid vigor”. Pangenomics can play an important role in unraveling gene members and families contributing to heterosis, according to the proposed model (Figure 5). Thus, it is clear that based on the model proposed [105], a new gene and variant finding is essential to explaining and utilizing heterosis for crop improvement. Single reference genomes cannot be used for novel gene discovery. Zhao et al. [13] utilized divergent species of rice *O. sativa* and relative *O. rufipogon* to map the rice pangenome. Based on an analysis of 1529 rice accessions, the divergent 57 accessions along with nine popular cultivars were sequenced to assemble the rice pangenome. Extensive PAVs were found in rice accessions based on the assembled pangenome, which is a useful resource for further studies. Hirsch et al. [15] analyzed 503 maize inbred lines for understanding developmental transitions from juvenile to vegetation and then to reproduction. The authors found that 16.4% of representative transcript assemblies were observed in all lines, while 82.7% expressed in a subset of lines. This shows the limitation of using a single genome for transcript mapping and reveals the importance of pangenomics for molecular characterization of heterosis phenomenon.

### 7.6. Facilitating Taxonomic Identification

Pangenomics is a useful tool to identify a species as well as gain a detailed understanding of its lifestyle habit and habitat. Species identification is important for various reasons, including diagnostics. Rouli et al. [106] describe an interesting case whereby the distinct morphological and biochemical features of *E. coli* and *Shigella* species led to their differential categorization. However, despite a myriad of differences, the mechanism of pathogenicity in *Shigella* and *E. coli*, particularly the enterohaemorrhagic invasive *E. coli* EIEC is identical as both enter epithelial cells to cause local inflammation leading to ulceration of the colon. The authors argue that *Shigella* and *E. coli* should be grouped together and that their distinction and individualization were due to medical diagnosis. Indeed, based on pangenomics studies, and based on cluster analysis using Clusters of Orthologous Groups of proteins (COGs) and Kyoto Encyclopedia of Genes and Genomes (KEGG), *Shigella* was found to be distributed among the different *E. coli* clusters. The principal component analysis revealed two clusters, both of which contain a mix of *E. coli* and *Shigella* species. It has also been observed that the lifestyle of a micro-organism also influences the type of genome that it contains. Microbes can exist in two states of lifestyle: allopatry (living alone in an environmental niche) and sympatry (living in a large community in an environmental niche). The allopatric microbes tend to have closed pangenomes, while those in sympatric lifestyles, have open pangenomes. Sympatric microbes gain genes to survive in diverse niches, while allopatric microbes face gene loss. This indicates the complementation of gene gain and loss events for the pangenome. Hence, the nature of pangenome can also indicate the lifestyle habit of an organism. The pangenome nature reveals intricate details of a microbe’s interaction with the environment. It is intriguing to note that *B. anthracis* is a soil bacterium but still contains a closed pangenome. This is because it stays dormant in the form of a spore with minimal interactions with outside. On the other hand, *Legionella pneumonia* stays intracellularly in amoeba, but is in a metabolically active state and thus possesses an open pangenome.

### 7.7. Strengthening Proteogenomics

In terms of functional annotation, a pangenome may also be looked upon in the form of pan-metabolome (complement of all metabolic reactions in a species), pan-regulon (collection of co-expressed genes), resistome (repertoire of all genes encoding for proteins that confer resistance to other organisms), etc. Pangenomes further improve our understanding of microbial species and can be utilized in proteogenomics-based identification of microbial flora in diverse biological samples. In this approach, the sample to be identified is taken, proteins are isolated and digested to result in a mix of peptides, which is specific for a particular species. This is referred to as Peptide Mass Fingerprint. Mass spectrometry has been used in the identification of microbial strains by analyzing the peptide fingerprint patterns of the sample proteins to proteomics databases. With the addition of new strains in the repositories, data mining becomes an issue as computational search becomes more and more demanding. Among the various techniques to accomplish peptide fingerprint matching, de Souza et al. [107] utilized computational algorithms to reduce the redundancy of protein databases of related bacterial species. This was denoted as MSMSpdbb (Multi-Strain Mass Spectrometry Prokaryotic DataBase Builder) (The Gade Institute, University of Bergen, Haukeland University Hospital, Bergen, Norway) approach. Given the increasingly more pangenomic data being generated, one way to allow robust microbial identification is to create customized databases where peptides from homologous proteins are not present in all the related bacterial strains.

Pangenomics keeps on adding new genomic information of new species. The information about Open Reading Frames (ORF) in the new genes, new features of homologous genes like differences in translational starting site (TSS) can be supplemented to existing protein databases to allow robust identification of biological samples. Caputo et al. [108] reported that while pangenomes identify novel strains like *Akkermansia muciniphila*, *Microvirga massiliensis*, etc., analysis of data like a discontinuity in the core/pangenome ratio can also indicate the presence of novel species. de Souza et al. [107] reported that concatenating protein sequences obtained from the pangenomics analysis of multiple organisms contained in public repositories leads to a thoroughly covered microbial sequence database, for sample identification. Thus, pangenomics contributes to microbial identification in multiple ways, including finding new strains, indicating the presence of other strains, and complementing protein sequence databases in public repositories.

### 7.8. Advancing Reverse Vaccinology

Vaccine development has witnessed paradigm shifts in the genomics and pangenomics era. Conventional vaccinology requires cultivable microorganisms, purification of components responsible for immunogenicity, immunogenicity testing in animal models, and the development of vaccines. However, there are disadvantages, as vaccines cannot be fabricated for non-culturable pathogens and the correlation of animal models with human subjects may not be high in certain cases. Moreover, only abundantly expressed antigens are generally tested. With the availability of pan-genomic sequences, virtually all antigens can be tested. The development of vaccines using genomic sequences has been referred to as reverse vaccinology, as its operating procedure is essentially the reverse of the steps taken in conventional vaccine development. There is no issue of the non-culturability of a pathogen for which genomic sequence has been made available. The proteins involved in host–pathogen interaction can be utilized for the prioritization of targets for vaccine development. The vast repertoire of applicable gene products, as revealed by pangenomics, have immense potential to develop specific vaccines for various pathogens and subtypes. Naz et al. [109] demonstrated the use of pangenomic data for vaccine development. The authors have designed a pipeline to scan the entire genomic complement of a pathogen to design effective vaccines against it. This approach termed as Pangenome-Reverse Vaccinology is a cost-effective technique to overcome the limitations associated with conventional vaccine development, by employing pangenomic DNA sequences. Dalsass et al. [110] reviewed the open-source platforms for bacterial vaccine antigen discovery. The shortcomings of the current prediction pipelines were highlighted. There is a need to expand the curation of protein datasets by incorporating negative results and inclusion of high-throughput secondary structure prediction methods like Circular Dichroism spectroscopy. This would enhance the prediction power for better translation in wet laboratory results.

## 8. Conclusions and Future Perspectives

Pangenomics has augmented both basic and applied research. It has contributed to a variety of interesting areas such as identification of industrially relevant microbial resources, vaccine designing, refining evolution and taxonomic identification, proteogenomics, deeper knowledge about host–pathogen interaction, and genetic makeup of important agronomic phenotypes. There remains an immense scope in pangenomics for understanding complex biological phenomena. Further refinements in core and accessory genome characterization leverage understanding of crop adaptation. It will also aid the discovery of new variations through novel haplotypes and functional molecular markers which will facilitate trait introgression or genomics-assisted breeding. This is expected to result in better utilization of heterosis for breeding improvement. Further, the development of portable sequencing technologies like Oxford Nanopore and integration of more robust and open-source high throughput visualization tools, along with efficient storage and retrieval of huge pangenomics data, would lead to progressive deployment of pangenomics for addressing different scientific issues in a cost-effect way by researchers.

## Figures and Tables

**Table 1:** Software/tools for pangenome analysis.

Software/Tool	Description/Role	URL Link	References
PanSeq	Extract the regions unique in the genome, Identify the SNPs and construct the file for phylogeny programme.	https://lfz.corefacility.ca/panseq/ (accessed on 17 September 2021)	[42]
PanFunPro	Homology detection and pairwise genome analysis in pan/core genome.	https://zenodo.org/record/7583#.YTR36p0zY2w (accessed on 17 September 2021)	[43]
GET_ HOMOLOGUES	Clustering proteins and nucleotide sequence into homologous group and analysis of overlapping sets of proteins	http://www.eead.csic.es/compbio/soft/gethoms.php (accessed on 17 September 2021)	[44]
ITEP	It is use for sequence alignment, metabolic, clustering, and protein prediction	https://price.systemsbiology.net/itep (accessed on 17 September 2021)	[45]
PanGP	Use for large-scale bacterial pangenome profile analysis with sampling algorithms.	https://pangp.zhaopage.com/ (accessed on 17 September 2021)	[46]
PGAP	Detection of homologous genes, orthologous genes, SNP, phylogenetic studies, pangenome plotting and functional annotation.	http://pgap.sf.net (accessed on 17 September 2021)	[47]
PGAT	To compare the gene content and sequence across multiple microbial genomes to identify the SNPs.	http://nwrce.org/pgat (accessed on 17 September 2021)	[48]
EDGAR	EDGAR performs homology analyses with a specific cutoff, Venn diagrams and interactive synteny plots.	https://bio.tools/edgar_genomics (accessed on 17 September 2021)	[49]
Micropan	This allows integration of pangenome and additional analyses within a single programming language environment	Package “micropan” in r software (accessed on 17 September 2021)	[50]
SplitMem	A graphic software for pangenome analysis software by de Bruijn graph.	https://sourceforge.net/projects/splitmem/ (accessed on 17 September 2021)	[51]
ClustAGE	Focused on the accessory genomic dimension of pangenome	http://vfsmspineagent.fsm.northwestern.edu/cgi-bin/clustage.cgi (accessed on 17 September 2021)	[52]
DeNoGAP	Help in gene prediction, protein classification and orthology search	https://github.com/DSGlab/DeNoGAP (accessed on 17 September 2021)	[53]
EUPAN	This was first to analyze eukaryotic pangenomes to identify core and accessory gene datasets	http://cgm.sjtu.edu.cn/eupan/index.html (accessed on 17 September 2021)	[30]
Harvest	This is useful for the analysis based on three modules Parsnp (core-genome analysis), Gingr (output visualization), and Harvest Tools (meta-analysis)	https://www.cbcb.umd.edu/software/harvest (accessed on 17 September 2021)	[54]
LS-BSR	Calculates a score ratio per coding sequence within a pangenome dataset using BLAST	https://github.com/jasonsahl/LS-BSR (accessed on 17 September 2021)	[55]
NGSPanPipe	Identify pangenome from short reads and output is compatible with other pangenome analysis tools	https://github.com/Biomedinformatics/NGSPanPipe (accessed on 17 September 2021)	[56]
PanACEA	Identification of genomic regions those are phylogenetically dissimilar.	https://github.com/JCVenterInstitute/PanACEA (accessed on 17 September 2021)	[57]
PanCake	Useful for clustering homologous genes and analyzing core/accessory genome	https://pypi.org/project/pancake/ ( accessed on 17 September 2021)	[58]
PanGeT	Pangenome analysis based on comparison at genome and proteome levels.	http://pranag.physics.iisc.ernet.in/PanGeT/ (accessed on 17 September 2021)	[59]
PanGFR-HM	Genomic/functional diversity and phylogenetic on genome-based between human associated microbial genomes	http://www.bioinfo.iicb.res.in/pangfr-hm/ (accessed on 17 September 2021)	[60]
PANINI	For rapid online visualization and analysis of the core and accessory genome evolutionary signal.	http://panini.pathogen.watch (accessed on 17 September 2021) and code at http://gitlab.com/cgps/panini (accessed on 17 September 2021)	[61]
PANNOTATOR	To ensure quality and standards for functional genome annotation among different strains	http://bnet.egr.vcu.edu/iioab/agenote.php (accessed on 17 September 2021)	[62]
PanOCT	PanOCT is a graph-based ortholog clustering tool of closely related prokaryotic genomes.	ftp://ftp.ncbi.nih.gov/blast/executables/release/ (accessed 17 September 2021)	[63]
Pan-Tetris	An interactive and dynamic visual inspection of gene occurrences in a pangenome table.	http://bit.ly/1vVxYZT (accessed on 17 September 2021)	[64]
PanTools	Annotating pangenomes, sequences adding, grouping genes, retrieving genomic regions and querying pangenome	http://www.bif.wur.nl (accessed on 17 September 2021)	[65]
PanViz	It can visualize from range of data formats of pangenomic data and mapping genes from existing pangenome.	https://github.com/thomasp85/PanViz (accessed on 17 September 2021)	[66]
PanWeb	It is a graphical interface of pangenome analysis generated from PGAP software.	http://www.computationalbiology.ufpa.br/panweb (accessed on 17 September 2021)	[67]
PanX	This tool identifies orthologous gene clusters in pangenomes, visualization, presence/absence pattern and identify SNPs	https://pangenome.org/ (accessed on 17 September 2021)	[68]
PGAdb-Builder	This is used to constructs a pangenome allele database (PGAdb).	http://wgmlstdb.imst.nsysu.edu.tw/ (accessed on 17 September 2021)	[69]
PGAP-X	Genome diversity and visualize genome structure and gene content to understand the evolution.	http://pgapx.ybzhao.com/ (accessed on 17 September 2021)	[22]
Piggy	Detection of highly divergent (“switched”) intergenic regions (IGRs) upstream of genes in pangenome	https://github.com/harry-thorpe/piggy (accessed on 17 September 2021)	[70]
Pyseer	This is helpful in genome-wide association studies in the microbes to identify potential genetic variation.	https://github.com/mgalardini/pyseer (accessed on 17 September 2021)	[71]
Seq-seq-pan	For sequential alignment of sequences to build a pangenome data structure and a whole-genome alignment.	https://gitlab.com/rki_bioinformatics (accessed on 17 September 2021)	[72]
Spine and AGEnt	Spine, find core-genome from a group of genomic sequences and AGEnt, find the accessory genome in draft genomic sequences	http://vfsmspineagent.fsm.northwestern.edu/index_age.html (accessed on 17 September 2021)	[73]
BPGA	Pangenome profile analysis, pangenome sequence extraction, exclusive gene family analysis, atypical GC content analysis and species phylogenetic analysis.	http://sourceforge.net/projects/bpgatool/ (accessed on 17 September 2021)	[74]
BGDMdocker	For pangenome analysis, visualization, clustering and genome annotation.	https://www.docker.com/whatisdocker (accessed on 17 September 2021)	[75]
PAN2HGENE	To identify new products, resulting in altering the α value behavior in the pangenome without altering the original genomic sequence.	https://sourceforge.net/projects/pan2hgene-software (accessed on 17 September 2021)	[76]
PATO	Core-genome and accessory genome identification and help to characterize population structure, annotate pathogenic features and create gene sharedness networks.	https://github.com/irycisBioinfo/PATO (accessed on 17 September 2021)	[77]
Panakeia	It analyses synteny and multiple structural patterns of the pangenome, help for biological diversity and evolution studied.	https://github.com/BioSina/Panakeia (accessed on 17 September 2021)	[78]
HUPAN	It is developed for pangenome analysis for humans/mammals	http://cgm.sjtu.edu.cn/hupan/ (17 September 2021) and https://github.com/SJTU-CGM/HUPAN (accessed on 17 September 2021)	[79]

## Data Availability

Not applicable.
